# Controversies over the mechanisms underlying the crucial role of the left fronto-parietal areas in the representation of tools

**DOI:** 10.3389/fpsyg.2013.00727

**Published:** 2013-10-10

**Authors:** Guido Gainotti

**Affiliations:** ^1^Department of Neurosciences, Center for Neuropsychological Research, Policlinico ‘A. Gemelli’, Università Cattolica del Sacro CuoreRome, Italy; ^2^Department of Clinical and Behavioral Neurology, Istituti di Ricovero e Cura a Carattere Scientifico Fondazione Santa LuciaRome, Italy

**Keywords:** tools representation, left fronto-parietal areas, embodied cognition theory, domains of knowledge hypothesis, innate theories, sensory-motor experiences, sensory-motor model of conceptual knowledge

## Abstract

Anatomo-clinical and neuroimaging data show that the left fronto-parietal areas play an important role in representing tools. As manipulation is an important source of knowledge about tools, it has been assumed that motor activity explains the link between tool knowledge and the left fronto-parietal areas. However, controversies exist over the exact mechanisms underlying this relationship. According to a strong version of the “embodied cognition theory,” activation of a tool concept necessarily involves re-enactment of the corresponding kind of action. Impairment of the ability to use tools should, therefore, lead to impairment of tool knowledge. Both the “domains of knowledge hypothesis” and the “sensory-motor model of conceptual knowledge” refute the strong version of the “embodied cognition hypothesis” but acknowledge that manipulation and other action schemata play an important role in our knowledge of tools. The basic difference between these two models is that the former is based on an innate model and the latter holds that the brain’s organization of categories is experience dependent. Data supporting and arguing against each of these models are briefly reviewed. In particular, the following lines of research, which argue against the innate nature of the brain’s categorical organization, are discussed: (1) the observation that in patients with category-specific disorders the semantic impairment does not respect the boundaries between biological entities and artifact items; (2) data showing that experience-driven neuroplasticity in musicians is not confined to alterations of perceptual and motor maps but also leads to the establishment of higher-level semantic representations for musical instruments; (3) results of experiments using previously unfamiliar materials showing that the history of our sensory-motor experience with an object significantly affects its neural representation.

## INTRODUCTION

Tools constitute a very important and very specific category of objects, which includes the man-made artifacts that have driven the transition from the prevalence of biological, innate factors to the prevalence of cultural determinants in the development of the human mind (Vaesen, 2012; Lefebvre, 2013). For this reason, the problem of specific aspects of the cognitive and neural bases of human tool use must be viewed in the context of the wider problem of the cognitive and neural bases of categories in general. In the l980s, Warrington and colleagues laid the groundwork for a contemporary approach to this problem. In an influential series of papers, these authors showed that different brain lesions can provoke different kinds of category-specific disorders which selectively affect action names/verbs (Baxter and Warrington, 1985; McCarthy and Warrington, 1985), biological entities (Warrington and Shallice, 1984; McCarthy and Warrington, 1991), and man-made artifacts (Warrington and McCarthy, 1983,^1987^). However, to explain these category-specific disorders they did not claim that various categories of knowledge are separately represented at the brain level. Rather, they proposed a general principle (i.e., the “differential weighting hypothesis”), which, on one side, acknowledges that concepts are based on the convergence of different perceptual and motor information in specific cortical areas but, on the other side, stresses the different weight that various sources of knowledge can have in the acquisition of different conceptual categories. According to this principle, category-specific semantic disorders result from disruption of the brain structures underlying perceptual, motor and language-related sources of knowledge, which have a critical role in organizing the corresponding categories. Within this context, category-specific disorders for verbs are considered due to disruption of the semantic aspects of actions and the dissociation (in objects) between living beings and artifacts is considered the consequence of the different weight of visual-perceptual and functional attributes in the construction of living and artifact categories (the “sensory-functional theory”). More specifically, this model suggests that the brain networks which are damaged in category-specific semantic disorders for animal and plant-life items have a critical role in processing the high-level visual attributes which allow distinguishing members of the “biological” categories. On the other hand, the cortical areas that are disrupted in patients with a prevalent defect for tools and other artifacts are involved in processing the functional and manipulative functions on which the knowledge of artifacts is based (Gainotti, 2000,^2005^; Buxbaum and Saffran, 2002; Buxbaum et al., 2007).

Warrington and colleagues’ positions were at variance with cognitive models (e.g., Pylyshyn, 1973; Fodor, 1975; Caramazza et al., 1990; Patterson and Hodges, 2000) that proposed the existence of a unitary, abstract and amodal semantic system. The latter was accessed by the highest levels of the various perceptual modalities (i.e., “structural descriptions”), which include a complete perceptual specification of objects prior to their meaningful recognition. According to these cognitive models, there should be no trace of the various sensory-motor modalities beyond the level of the corresponding “structural descriptions,” because the format of semantic representations should be symbolic, abstract, amodal and propositional. On the other hand, some reviews of the anatomical correlates of category-specific disorders for living beings and artifacts were consistent with Warrington and colleagues’ interpretations: first, those by Saffran and Schwartz (1994) and by Gainotti et al. (1995) and, subsequently, in a more detailed manner, by Gainotti (2000) and Capitani et al. (2003). Indeed, all these reviews showed that in patients with category-specific semantic disorders for living beings lesions bilaterally affect the anterior parts of the temporal lobes, where the ventral stream of visual processing terminates (Ungerleider and Mishkin, 1982; Mishkin et al., 1984; Goodale et al., 1991); but in patients with impaired knowledge of tools and other artifacts, the lesions encroach upon the inferior parts of the left frontal and parietal lobes, which process action and somato-sensory data. The importance of the inferior parts of the left frontal and parietal lobes in tool representation was supported by the anatomo-clinical data of Buxbaum et al. (2000) and Buxbaum and Saffran (2002) and the functional magnetic resonance imaging (fMRI) experiments conducted by Kellenbach et al. (2003) and Boronat et al. (2005). The first authors showed that the expression “functional attributes,” which should characterize artifacts, includes heterogeneous components. They, indeed, distinguished the function of an object from its manipulation and suggested that because “manipulation” is related to a sensory-motor activity it might be the component most tightly linked to the “differential weighting hypothesis.” Kellenbach et al. (2003) and Boronat et al. (2005) confirmed this hypothesis by asking normal subjects in two fMRI studies to make judgments about actions and functions associated with manipulable and non-manipulable objects. Both studies showed that the left inferior frontal and parietal areas responded more strongly to actions (vs functions) and to manipulable (vs non-manipulable) objects. Therefore, these results confirmed that brain regions specialized for sensory-motor functions have a critical role in the representation of tools and other manmade objects. This, obviously, does not mean that tools are represented only in action linked left fronto-parietal cortical areas, because some authors (e.g., Lewis, 2006 and Frey, 2007) have rightly noted that this system must interact, within a “tool use network,” with an other more general system, involved in conceptualizing, planning, and accessing knowledge associated with tool use. According to Frey (2007), this interaction should involve, on one hand, sensory-motor knowledge, represented within the dorsal stream of visual processing (Goodale et al., 1991) and, on the other hand, semantic knowledge, represented, at least in part, within the ventral stream. An alternative model, advanced by proponents of the “Semantic Hub” hypothesis (e.g., Patterson et al., 2007; Lambon Ralph and Patterson, 2008), assumes that this more general semantic network should be bilaterally located in the anterior portions of the temporal lobes, which are atrophic in Semantic dementia (SD). Hodges et al. (2000) have indeed, shown that in patients with SD naming, semantic knowledge and use of tools can be markedly impaired. The present review, however, will not dwell on this problem, because it will be focused on the specific issue of the mechanisms underlying the crucial role of the left fronto-parietal areas in the representation of tools and not on the general problem of the cortical network underlying tools representation. As a matter of fact, according to the “Semantic Hub” hypothesis, a bilateral atrophy of the anterior temporal lobes should provoke a semantic impairment, more or less equally affecting all kinds of concepts. No specific interaction should, therefore, be predicted between the “semantic hub” and the specific left fronto-parietal cortical representation of the tools category.

Controversies over the mechanisms underlying the crucial role of the left fronto-parietal areas in the representation of tools can be viewed, from a very general point of view, as a “tool” specific version of some of the oldest controversies in neuroscience: that between nature and nurture as well as that between localization principle, emphasizing the specificity and modularity of the brain on the one hand and holistic views, stressing unified, global functions and Gestalt phenomena on the other hand (see also Edelman, 1993 and Tononi et al., 1998). The present review highlights strength and weaknesses of three accounts which have tried to explain the relationships between tool knowledge, manipulation and left frontal and parietal areas, following the lines of thought illustrated in this introduction.

## MODELS ADVANCED TO ACCOUNT FOR THE RELATIONSHIP BETWEEN MANIPULATION AND TOOL KNOWLEDGE

Different theoretical models have been advanced to explain the relationship between manipulation and tool knowledge and the role played by the left ventral frontal and parietal areas. One of these interpretations is based on a strong version of the “embodied cognition hypothesis” (Barsalou, 1999; Barsalou et al., 2003; Gallese and Lakoff, 2005) and maintains that the conceptual processing of tools necessarily involves the retrieval or simulation of the movements associated with tool usage. According to these views, motor programs are run in the course of object recognition and are necessary to ground conceptual knowledge of objects. One prediction that can be made on the basis of this hypothesis is that loss or impairment of motor programs concerning the use of tools should be associated with disruption of the corresponding conceptual tool knowledge. This “strong version,” however, is not the only account of the embodied cognition theory, because a weaker version, simply stressing the importance of manipulation and other action schemata in our knowledge of tools, is definitely accepted by most authors and because some representatives of the embodied cognition theory (e.g., Barsalou, 2008), seem to separate themselves from the rigid view that has been just described.

Other theoretical models therefore acknowledge that motor programs associated with tool use have an important role in the construction of tool representation, but deny that a necessary and sufficient relationship exists between the re-enactment of these sensory-motor processes and tool knowledge.

One of these models is the “domains of knowledge” hypothesis, proposed by Caramazza (1998); Caramazza and Shelton (1998), Capitani et al. (2003) and Caramazza and Mahon (2003). This model acknowledges that conceptual knowledge is organized in categories at the brain level and holds that innate (rather than experience-dependent) factors subsume this categorical organization. It also assumes that natural selection produced specialized and therefore dissociable neural circuits for animals, “fruit and vegetables,” tools and “conspecifics,” because these categories have an important and specific role in human survival (Caramazza and Mahon, 2003). One development of this model, called “the distributed domain-specific hypothesis” (Mahon and Caramazza, 2011), argues that innately determined patterns of connectivity mediate the integration of information critical for the organization of each domain of knowledge.

A different theoretical model, which acknowledges the important (but not exclusive) role of specific motor programs in the construction of tool representation, is the “sensory-motor model of conceptual knowledge” (Saffran and Schwartz, 1994; Gainotti et al., 1995; Chao et al., 1999; Chao and Martin, 2000; Gainotti, 2000,^2005^; Martin et al., 2000; Martin and Chao, 2001; Martin, 2007). This model holds that various perceptual, motor and encyclopedic sources of knowledge have different weights in the construction of different living and artifact categories and attributes their role to experience-dependent (rather than innate) factors. Data supporting and contrasting each of these models will be briefly discussed in the following sections of this review.

### DATA SUPPORTING AND CONTRASTING THE “STRONG” VERSION OF THE “EMBODIED COGNITION THEORY”

As a general rule, data supporting the “strong” version of the “embodied cognition theory” come from functional neuroimaging experiments, whereas data weakening or undermining this theory come from the field of brain pathology. Several authors have documented that the left inferior frontal and parietal areas are selectively activated when subjects perform tasks with tool stimuli but not with non-manipulable objects (see Grèzes and Decety, 2001; Martin and Chao, 2001 and Caramazza and Mahon, 2006 for reviews). Other authors (e.g., Hauk et al., 2004; Buccino et al., 2005; Tettamanti et al., 2005; Kemmerer et al., 2008; Pulvermuller et al., 2009; Arévalo et al., 2012) have shown that a more fine grained relationship exists between actions performed with tool stimuli and activation of specific frontal and parietal areas. Indeed, when normal subjects are presented with stimuli involving actions that refer to specific body parts (such as objects associated with the use of the hand, mouth or foot), activation prevails in the corresponding somatotopically organized cortical areas. Therefore, the activation peak for each effector corresponds with the somatotopic organization of the motor homunculus, which was first described by Penfield and Boldrey (1958).

Critics of these findings (e.g., Fischer and Zwaan, 2008; Arévalo et al., 2012) noted that: (a) functional neuroimaging evidence showing that motor programs participate in verbal and non-verbal tool knowledge does not imply that these action schemata are necessary or sufficient to support tool processing; (b) the somatotopical distribution of activations observed in the fMRI studies is not always clear and a good match for all three effectors across tasks is rarely reported (Fernandino and Iacoboni, 2010; Kemmerer and Gonzalez-Castillo, 2010).

Furthermore, lesion data, which are more relevant to clarify whether the activated areas are necessary for grounding tool conceptual knowledge or simply participate in their processing, have provided results inconsistent with the strong version of the “embodied cognition theory.” Thus, research on patients with apraxia, whose performance is impaired when imitating observed actions, using objects or pantomiming their use from visual presentation, has shown that the ability to use objects may be differentially impaired relative to naming objects or knowing their function (Buxbaum et al., 2000; Buxbaum and Saffran, 2002; Rosci et al., 2003; Negri et al., 2007). Garcea et al. (2013) reported the detailed study of a patient with a large left hemisphere lesion whose object knowledge was relatively spared in spite of a severe motor (action production) defect and impaired conceptual knowledge of actions. Arévalo et al. (2012) presented left hemisphere stroke patients with pictures and words representing objects and actions typically associated with use of the hand, mouth and foot. They correlated results obtained on these tasks with data obtained from voxel-based lesion-symptom mapping analyses, but found no support for a correlation between body parts involved in the use of objects and somatotopically organized locus of damage. Taken together, the few studies that have used lesion data to test predictions deriving from the “embodied cognition theory” have provided data inconsistent with this theory. In keeping with these conclusions, based on the comparison between results of functional neuroimaging experiments and of anatomo-clinical studies, are also more general considerations. If we take into account, for instance, the automatic perception of object affordances (namely the fact that the action representations of an object can be automatically activated from its view) we must acknowledged that this automaticity is not consistent with the attainment of tool identity from action representations (Creem-Regehr and Lee, 2005). Contrasting opinions exist, however, on this subject. Some authors (e.g., Bub et al., 2008) suggest that that activation of motor representations depends on a form of attentional orienting to the object. Other authors (e.g., Randerath et al., 2013) propose that there are limitations to the automatic perception of affordances, because factors such as tool use context, and type of task play an influential role. Finally, it must be noted that the “strong version” of the embodied cognition theory entails a none or all mechanism, which render the model rather implausible.

### DATA SUPPORTING “INNATE” AND “EXPERIENCE-DEPENDENT” INTERPRETATIONS OF THE RELATIONSHIPS BETWEEN MANIPULATION AND TOOL KNOWLEDGE

Both the “domains of knowledge hypothesis” and the “sensory-motor model of conceptual knowledge” refute the strong version of the “embodied cognition hypothesis” but acknowledge that manipulation and other action schemata have an important role in our knowledge of tools. The basic difference between these two models is that the “domains of knowledge hypothesis” is based on an innatist model and the “sensory-motor model of conceptual knowledge” maintains that the brain’s organization of categories is experience-dependent.

In fact, the “domains of knowledge” hypothesis’ holds that the brain is really organized by categories and that this organization results from innately determined patterns of connectivity which mediate the integration of information critical for each category. On the contrary, the “sensory-motor model of conceptual knowledge” holds that the categorical organization of the brain is only apparent because each category results from the convergence of different sources of knowledge whose organization is not innate but experience-dependent. According to the first model, which was labeled “the distributed domain-specific hypothesis” by Mahon and Caramazza (2009), a domain-specific neural system is a network of brain regions in which each region processes a different type of sensory, motor, affective or conceptual information about the same category of objects. Furthermore, the computations that must be performed on items in the same category are sufficiently specific to merit a specialized process. For instance, there is a strong need to integrate motor-relevant information with visual information for tools and other artifacts; this need is less strong for animals and faces. In a similar manner, there is a strong need to integrate affective information, biological motion processing and visual form information for animals and conspecifics; this need is less strong for tools and other artifacts. Thus, supporters of the “distributed domain-specific hypothesis” propose that specialization for faces in the lateral fusiform area of the ventral visual stream occurs because this region of the brain is connected with the amygdale and the superior temporal sulcus, which are important for the extraction of socially relevant information. By contrast, specialization for tools and manipulable objects is driven by connectivity between the inferior frontal and parietal cortex, which subserve object manipulation and regions of the medial fusiform gyrus, which are involved in tools visual processing. Data supporting the innate nature of these patterns of connectivity come from work indicating that congenitally blind subjects show activation for words (presented in Braille) in the same regions of the ventral stream activated by visually presented words in sighted individuals (Buchel et al., 1998). Furthermore, Mahon et al. (2009) showed that the same medial-to-lateral bias in category preferences for artifacts vs animals which is present in the ventral surface of the temporo-occipital cortex in sighted individuals is also present in congenitally blind subjects. Mahon and Caramazza (2011) suggested that if visual experience is unnecessary for the emergence of category-specificity in the ventral stream, innate connectivity between regions of the ventral stream and other regions of the brain could drive category-specificity.

Nevertheless, some data argue against the “domains of knowledge hypothesis” and the innate nature of the brain’s categorical organization. Among these, we can include the following clinical and experimental data:

(a) The observation that in patients with category-specific disorders the semantic impairment does not respect the boundaries between living/biological entities and non-living/artifact items. In particular, Warrington and Shallice (1984); Warrington and McCarthy (1987), Basso et al. (1988); Silveri and Gainotti (1988); Damasio (1990), Hillis and Caramazza (1991); Sacchett and Humphreys (1992), Breedin et al. (1994); Farah et al. (1996), Forde et al. (1997), Dixon et al. (2000) and Masullo et al. (2012) showed that the representation of “body parts” tends to be disrupted in association with that of artifacts, and the representation of “musical instruments” tends to be disrupted in association with that of biological entities. For two reasons, this observation is consistent with the “sensory-functional theory” and inconsistent with the “domains of knowledge hypothesis.” On one side, we observe here a systematic breakdown across categories. On the other side, musical instruments (which are not recognized by their function but by their different shape and acoustic features) are more similar to “living” items than to other artifacts from the viewpoint of their sources of knowledge, whereas body parts are identified on the basis of the somato-sensory and action-related information, which also has a critical role in the recognition of tools and other artifacts (Gainotti et al., 2009).
(b) Still within the category of musical instruments (but shifting from the contrast between the disruption of real categories and the disruption of representations based on the same sources of knowledge to the “innate vs experience-dependent” opposition), interesting data supporting the experience-dependent interpretation were recently reported by Hoenig et al. (2011). These authors, starting from the premise that professional musicians constitute a very good model for understanding experience-dependent plasticity in the human brain, wondered whether this neuroplasticity might extend beyond basic perceptual and motor functions and shape the semantic representation of musical instruments. Using fMRI, they showed that in musicians (but not in musical laypersons) conceptual processing of visually presented musical instruments activates the auditory association cortex encompassing the right posterior superior temporal gyrus, which is also recruited in the auditory perception of real sounds. Therefore, experience-driven neuroplasticity in musicians is not confined to alterations of perceptual and motor maps but also leads to the establishment of higher-level semantic representations for musical instruments.
(c) The role of prior motor experience in the cortical representation of objects was also addressed by Creem-Regehr et al. (2007); Kiefer et al. (2007), Weisberg et al. (2007) and Bellebaum et al. (2013). As the history of previous sensory-motor experience with familiar objects cannot be controlled, these authors tried to use previously unfamiliar material and submitted their subjects to different types of extensive training with these objects. In Kiefer et al.’s (2007) study, the plasticity of conceptual representations was assessed by training subjects with novel objects under different training conditions. In one class of stimuli, object categorization was based on a detail feature of the novel objects, affording a particular action. During training, participants were asked either to make an action pantomime toward the detail feature or simply to pay attention to it, by pointing to it with their index finger. Only in the pantomime group an early activation was found in the frontal areas, whereas in the pointing training group this effect was absent. These results show that action information contributes to conceptual processing, depending on the specific learning experience, and suggest that conceptual representations are established by the learning-based formation of cell assemblies in different cortical areas.

Creem-Regehr et al. (2007) investigated, by means of fMRI, the influence of action knowledge associated with viewing, grasping, and using novel graspable objects. Participants were trained on complex actions associated with novel objects (“tools”) and had experience manipulating other visually similar novel objects (“shapes”). The largest differences between “tools” and “shapes” were found in using, in which greater effect sizes were observed for tools versus shapes in the left inferior parietal lobule (IPL), the pre-supplementary motor cortex (pre-SMA) and, marginally, in the left ventral premotor cortex (VPM). These results suggest that representations of tools are constructed on the basis of complex action schemata, which recruit processes related to graspability, action plans and use of objects.

Weisberg et al. (2007) used fMRI in subjects who should visually match pictures of novel objects before and after extensive training dealing with the use of these objects to perform specific tool-like tasks. After training, neural activity emerged in regions associated with the motion (left middle temporal gyrus) and manipulation (left intraparietal sulcus and premotor cortex) of common tools, showing that experience of direct interaction with previously unfamiliar objects led to new neural object representations in the same cortical areas underlying the neural representation of tools. Finally, Bellebaum et al. (2013) studied with fMRI the impact of different types of object-related sensorimotor experiences on the neural representations of novel objects, contrasting the manipulation training (MTO) with the visual training (VTO) and the absence of training (NTO). The post-training activity in the left inferior/middle frontal gyrus and the left posterior IPL was higher for MTO than for VTO and NTO suggesting that manipulation experience specifically yields higher activities in regions of the fronto-parietal cortex.

(d) The final point, which argues against the hypothesis that an innate connectivity pattern may subsume the categorical organization of the human brain, is that handedness, rather than hemispheric language lateralization, seems to account for the special role played by the left ventral frontal and parietal areas in tool knowledge. In the introductory part of this review, I mentioned that in category-specific semantic disorders for living beings lesions affect the anterior parts of the temporal lobes bilaterally (where highly processed visual data are integrated with other sensory modalities). Differently, in patients with impaired knowledge of tools and other artifacts, lesions encroach upon the inferior parts of the left frontal and parietal lobes, which process action and somatosensory data. A theory stressing the innate aspects of brain organization and a theory stressing the importance of experience-dependent factors should make opposite predictions about the relationships among lateralization of the tool-related fronto-parietal activation, language lateralization and handedness. Innate theories should predict that strongly left-handed subjects will continue to show left fronto-parietal activation because of the same genetic factors which subsume the left hemisphere specialization for language (Annett, 2000; Corballis, 2009). Experiential theories should predict right fronto-parietal activation resulting from the execution of movements with the left side of the body. Two recent studies were conducted by Lewis et al. (2006) and Willems et al. (2010) in strong right- and left-handers to evaluate the role played by asymmetries in motor experience and the left dominance for language on the lateralization of tool representation. In the first study, Lewis et al. (2006) compared the pattern of cortical activation evoked by hand-manipulated tool sounds and animal vocalizations and found that tool sounds preferentially evoke activity in high-level motor-related cortical regions of the hemisphere opposite to the dominant hand. In the second study, Willems et al. (2010) used fMRI to compare premotor activity associated with understanding action verbs (strictly related to tool use) and showed that right-handers preferentially activated the left premotor cortex and left-handers, the right premotor areas. Therefore, in both studies and in agreement with the positions defended by the theory stressing the importance of experience-dependent factors, the laterality of cortical regions activated by high-level action and tool use was related to the side of the body involved in actions and not to left-hemisphere dominance for language. Note, however, that in a paper recently published by Goldenberg (2013) on “apraxia in left-handers” there were three aphasic patients with pervasive apraxia caused by left-sided lesions, who showed a dissociation of apraxia from handedness. Conversely there were also three patients with pervasive apraxia caused by right brain lesions without aphasia, who showed a dissociation of apraxia from aphasia. The implications of these data for the problem at issue requires clarifications.

## CONCLUSION

Taken together, results of the present review suggest that neither a strong version of the “embodied cognition theory” nor an “innate” categorical organization of conceptual knowledge can account for: (a) the important role of manipulation and other action schemata in our knowledge of tools and (b) the links between tool knowledge and the inferior fronto-parietal areas. On the other hand, the “sensorimotor model of semantic knowledge” can explain data obtained in brain-damaged patients (showing that tool knowledge can be spared after disruption of the motor processes engaged in tool use) and data stressing the role of prior motor experience in the construction of the cortical representation of objects. Furthermore, the sensorimotor model of semantic knowledge is supported by the results of studies that assessed the weight of various sources of knowledge in the construction of biological and artifact categories in normal subjects. These studies used either feature verification tasks (e.g., Vigliocco et al., 2004; McRae et al., 2005) or Likert scales (e.g., Gainotti et al., 2009,^2012^; Hoffman and Lambon Ralph, 2013) to evaluate the weight that different “sources of knowledge” could have in the construction of different semantic categories. Regardless of the methodology used in these investigations, results showed that visual information is considered the dominant source of knowledge across categories, but the second most important sources of information are different in biological and artifact categories. In fact, they consist of other perceptual data for the living categories and actions and somato-sensory data for tools and the other artifact categories. Therefore, vision, actions and somato-sensory information have a major role in the representation of tools and other artifacts, whereas visual and other perceptual input have a dominant role in the representation of animals and other living things. The fact that normal subjects have considered both vision and action-related information as important sources of knowledge about tools and other artifacts supports: (a) the crucial role of the left fronto-parietal areas, subsuming transitive actions, in the representation of tools; (b) the thesis of authors (e.g., Creem-Regehr and Lee, 2005; Buxbaum et al., 2007; Frey, 2007) who have claimed that both the dorsal and the ventral stream must play a role in the representation of tools.

It would certainly be desirable, at the end of this survey, to predict (if we assume that a “strong version” of the embodied cognition hypothesis is untenable), which are the future directions of research that could more strongly support the “domains of knowledge” or the “sensory-motor model of conceptual knowledge” hypothesis. However, a definite choice between “innatistic” and “experience dependent” mechanisms can hardly be made, because both mechanisms certainly intervene in the cognitive development. Coming back to the part of this survey, in which I claimed that tool-related research cannot be considered apart from investigations concerning in general the brain categorical organization, I think that, in any case, it should be important to more clearly assess: (a) if in category-specific disorders the semantic impairment respects or not the boundaries between biological entities and artifacts; (b) what is the role of the patient’s familiarity with disrupted and spared categories, to see if this variable can strongly influences the observed patterns of categorical semantic impairment.

## Conflict of Interest Statement

The author declares that the research was conducted in the absence of any commercial or financial relationships that could be construed as a potential conflict of interest.
